# Serum Creatinine Level at One Month Post-Transplant Predicts 3-Year and 5-Year Graft Survival and Renal Function: A Large Single-Center Retrospective Cohort Study

**DOI:** 10.3390/jcm15114238

**Published:** 2026-05-30

**Authors:** Jungjun Lee, Sunyoung Son, Manki Ju

**Affiliations:** Department of Surgery, Gangnam Severance Hospital, Yonsei University College of Medicine, Seoul 06273, Republic of Korea

**Keywords:** kidney transplantation, serum creatinine, graft survival, glomerular filtration rate, long-term outcomes, post-transplant monitoring

## Abstract

**Background**: Contemporary kidney transplantation increasingly includes high-immunologic-risk recipients, ABO-incompatible transplantation, deceased-donor transplantation, and desensitization protocols. Although early post-transplant serum creatinine has historically been associated with long-term graft outcomes, its prognostic value in modern high-risk transplant cohorts remains insufficiently characterized. We evaluated whether serum creatinine at one month post-transplant independently predicts 3-year and 5-year graft survival and renal function in a large contemporary kidney transplant cohort. **Methods**: Among 1895 consecutive KT recipients (2005–2018), patients were stratified by one-month serum creatinine into four quartiles: Q1 (<1.0 mg/dL, *n* = 398), Q2 (1.0–1.23, *n* = 480), Q3 (1.23–1.52, *n* = 487), and Q4 (≥1.52, *n* = 530). Primary endpoints were 3- and 5-year death-censored graft survival. **Results**: Median follow-up was 95 months. Three-year graft survival was 97.2%, 96.2%, 95.3%, and 90.3% for Q1–Q4, respectively; 5-year survival was 94.5%, 94.6%, 92.5%, and 86.9%. eGFR stratification persisted at both 3 and 5 years. After adjustment including donor and recipient BMI, Q4 creatinine independently predicted graft failure (HR, 1.590; 95% CI, 1.049–2.412; *p* = 0.029). One-month serum creatinine also remained significant as a continuous variable (HR, 1.409 per 1.0 mg/dL increase; 95% CI, 1.279–1.551; *p* < 0.001). ROC analysis identified an optimal cutoff of 1.39 mg/dL (AUC, 0.622; sensitivity, 52.6%; specificity, 65.0%). **Conclusions**: One-month serum creatinine is a robust and independent predictor of graft survival and renal function after kidney transplantation. These findings support its use as a simple early risk-stratification marker and as a trigger for targeted post-transplant surveillance.

## 1. Introduction

Kidney transplantation (KT) remains the optimal therapy for end-stage renal disease, conferring superior patient survival and quality of life compared with chronic dialysis [1,2]. Despite advances in immunosuppression and surgical techniques, progressive graft dysfunction remains an important clinical challenge, ultimately leading to graft loss [3].

Multiple donor- and recipient-related factors influence short- and long-term graft outcomes, including donor age, cold ischemia time [4], human leukocyte antigen (HLA) mismatching [5,6], delayed graft function (DGF), and calcineurin inhibitor (CNI) nephrotoxicity [7,8,9]. Acute rejection episodes, particularly within the first year, remain among the strongest modifiable predictors of graft loss [10,11,12].

Previous studies have established early graft function, assessed using serum creatinine or estimated glomerular filtration rate (eGFR) shortly after transplantation, as a strong predictor of long-term outcomes [13]. Analyses of early eGFR trajectories have shown that rapid functional decline during the first post-transplant months independently predicts graft loss [14], while machine learning models incorporating early time-point data provide incremental improvement over single time-point measures [15]. Nonetheless, serum creatinine-based eGFR remains superior to emerging biomarkers for discriminating long-term graft loss [16]. However, most prior studies were conducted before widespread adoption of contemporary high-risk transplantation protocols, including desensitization for ABO-incompatible recipients, plasmapheresis for antibody removal, and rituximab-based regimens [17]. The Korean transplant landscape has changed substantially since 2000, with marked increases in highly sensitized recipients, ABO-incompatible transplantation, and marginal deceased-donor utilization, highlighting the need to re-examine the predictive value of early serum creatinine in this modern context.

In our previous single-center study, serum creatinine at one-month post-transplant independently predicted long-term overall graft survival in a contemporary Korean KT cohort [18]. However, that study did not explicitly define 3-year and 5-year graft survival as primary endpoints, perform landmark analyses to examine whether the prognostic impact of early creatinine increases over time, or characterize serial eGFR trajectories at these clinically relevant milestones. Addressing these gaps has direct implications for post-transplant surveillance, patient counseling, and intensified interventions in high-risk patients.

In the present study, we analyzed a large single-center cohort to (1) compare 3-year and 5-year graft survival across four one-month post-transplant serum creatinine quartile groups; (2) describe serial eGFR trajectories at 1, 3, and 5 years; and (3) identify independent predictors of graft failure using multivariable Cox regression and landmark analyses at 3 and 5 years.

## 2. Materials and Methods

### 2.1. Study Design and Patient Population

This was an extended follow-up study of a previously published single-center retrospective cohort [18]. The original study reported that serum creatinine at one-month post-transplant independently predicted long-term graft survival. In the present analysis, we extended the follow-up duration and redefined the primary endpoints as 3-year and 5-year graft survival, with additional landmark analyses and serial eGFR trajectory assessments not included in the original report.

Consecutive KT recipients who underwent transplantation between January 2005 and December 2018 at a single Korean tertiary referral center were screened. Among initial 1976 KT procedures, 81 cases were excluded because of early primary graft failure within 30 days (*n* = 9), loss to follow-up within 3 months (*n* = 2), pediatric recipients or donors (age < 18 years, *n* = 37), or combined organ transplantation (*n* = 33). The final analytical cohort comprised 1895 recipients.

### 2.2. Serum Creatinine Quartile Groups

Recipients were stratified into four groups according to serum creatinine measured at one month post-transplant (30 ± 7 days): Q1 (<1.0 mg/dL, *n* = 398), Q2 (1.0–1.23 mg/dL, *n* = 480), Q3 (1.23–1.52 mg/dL, *n* = 487), and Q4 (≥1.52 mg/dL, *n* = 530). The quartile-based approach was chosen to avoid arbitrary threshold selection, maintain balanced group sizes, and assess a graded association between early graft function and long-term outcomes. Serum creatinine at one month was selected as the primary stratification variable because it is routinely measured, immediately available, and widely used during early outpatient follow-up. Serial eGFR was used for longitudinal assessment because it provides a standardized estimate of renal function that accounts for recipient demographic factors.

### 2.3. Outcomes

The primary endpoints were 3-year and 5-year death-censored graft survival. Secondary endpoints included serial eGFR, calculated using the four-variable Modification of Diet in Renal Disease (MDRD) equation, at 1, 3, and 5 years post-transplant, and the cumulative graft failure rate. Graft failure was defined as return to any dialysis modality or kidney retransplantation.

### 2.4. Immunosuppression Regimens

During the study period, immunosuppressive regimens evolved according to institutional practice and contemporary transplant protocols. Maintenance immunosuppression generally consisted of a calcineurin inhibitor, an antimetabolite, and corticosteroids. Tacrolimus-based therapy increased over time and became the predominant calcineurin inhibitor regimen, whereas cyclosporine use decreased. Mycophenolate-based antimetabolite therapy also became increasingly common. IL-2 receptor antagonist induction was widely used in standard-risk recipients, while anti-T/B-cell induction was selectively used in recipients with higher immunologic risk, delayed graft function, or retransplantation. For ABO-incompatible or sensitized recipients, desensitization strategies incorporating plasmapheresis and rituximab were increasingly adopted during the later study period.

### 2.5. Statistical Analysis

Continuous variables are presented as mean ± standard deviation (SD) and were compared using one-way analysis of variance (ANOVA) with post hoc Bonferroni correction. Categorical variables are expressed as frequency (percentage) and were compared using the chi-square test or Fisher’s exact test, as appropriate. Time-to-event outcomes were analyzed using Kaplan–Meier survival curves and compared using log-rank tests. Multivariable Cox proportional hazards regression was used to identify independent predictors of graft failure, including variables with *p* < 0.10 in univariable analysis. The proportional hazards assumption was verified using Schoenfeld residuals. Donor BMI and recipient BMI were additionally included in the multivariable Cox model in response to reviewer comments. One-month serum creatinine was also evaluated as a continuous variable. ROC analysis was performed to determine the optimal one-month serum creatinine cutoff for graft failure using the Youden index, and the resulting dichotomized variable was analyzed in an additional Cox model. Observation periods were compared across quartile groups using the Kruskal–Wallis test.

To address the time-dependent nature of the prognostic signal, landmark analyses were conducted at 3 and 5 years and restricted to recipients who had not experienced graft failure or death by each landmark time point. All analyses were performed using SPSS version 25.0 (IBM Corp., Armonk, NY, USA) and R version 4.2.0. Statistical significance was defined as two-sided *p* < 0.05.

## 3. Results

### 3.1. Baseline Characteristics

Baseline characteristics of the 1895 recipients stratified by serum creatinine quartiles are shown in Table 1. The cohort included 398, 480, 487, and 530 recipients in Q1, Q2, Q3, and Q4, respectively. *p*-values were added for all baseline variables. Higher serum creatinine quartiles were associated with older donor age, higher recipient BMI, greater deceased-donor utilization, higher rates of acute rejection within one year, and delayed graft function/acute tubular necrosis (all *p* < 0.05). Donor BMI did not differ significantly among groups (*p* = 0.057).

### 3.2. Serial eGFR Trajectories

**Figure 1:**
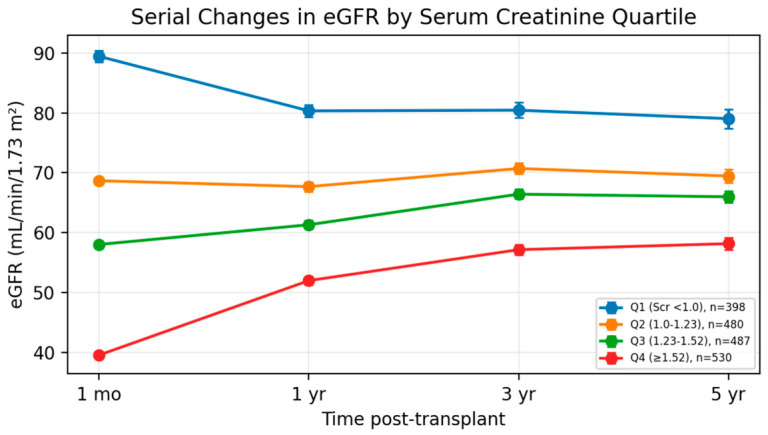
Serial changes in estimated glomerular filtration rate (eGFR; mean ± SE) by serum creatinine quartile from 1 month to 5 years post-transplant. Between-group differences were significant at all evaluated time points (*p* < 0.001, one-way ANOVA).

Serial eGFR values at 1 month, 1 year, 3 years, and 5 years post-transplant by quartile group are presented in Table 2 and illustrated in Figure 1. Mean eGFR differed significantly across groups at 1 month (Q1, 89.4 ± 19.6; Q2, 68.7 ± 10.2; Q3, 58.0 ± 8.6; Q4, 39.5 ± 13.2 mL/min/1.73 m^2^; *p* < 0.001), 1 year (Q1, 80.3 ± 19.3; Q2, 67.7 ± 17.2; Q3, 61.3 ± 15.2; Q4, 52.0 ± 15.0 mL/min/1.73 m^2^; *p* < 0.001), 3 years (Q1, 80.4 ± 23.8; Q2, 70.7 ± 19.1; Q3, 66.4 ± 16.2; Q4, 57.1 ± 17.3 mL/min/1.73 m^2^; *p* < 0.001), and 5 years (Q1, 79.0 ± 25.3; Q2, 69.4 ± 20.9; Q3, 66.0 ± 18.5; Q4, 58.2 ± 19.1 mL/min/1.73 m^2^; *p* < 0.001). Within Q4, 111 of 530 recipients (20.9%) had 1-month eGFR <30 mL/min/1.73 m^2^, whereas 419 (79.1%) had eGFR ≥30 mL/min/1.73 m^2^, indicating substantial variability within the highest creatinine quartile.

### 3.3. Three-Year and 5-Year Graft Survival

Graft survival data are summarized in Table 3. Over the entire follow-up period (median, 95 months; range, 0–189 months), 251 graft failure events were recorded, including 33 (8.3%), 52 (10.8%), 59 (12.1%), and 107 (20.2%) in Q1, Q2, Q3, and Q4, respectively. Kaplan–Meier analysis demonstrated progressively lower graft survival with increasing serum creatinine quartile at both the 3-year and 5-year landmarks.

**Figure 2:**
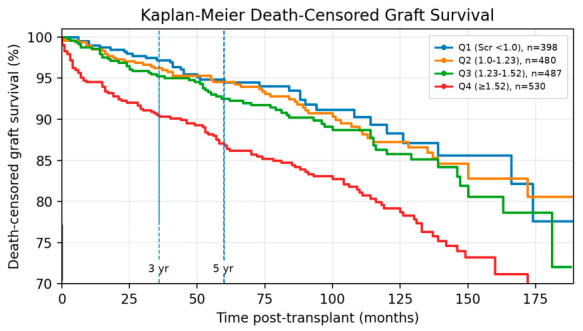
Kaplan–Meier death-censored graft survival curves stratified by serum creatinine quartile at one-month post-transplant. Dashed vertical lines indicate the 3-year and 5-year landmark time points.

The 3-year graft survival rates were 97.2%, 96.2%, 95.3%, and 90.3% for Q1 through Q4, respectively, and the corresponding 5-year rates were 94.5%, 94.6%, 92.5%, and 86.9%. The absolute difference in 5-year graft survival between Q1 and Q4 was 7.6 percentage points, reflecting a clinically meaningful prognostic gap attributable to early creatinine elevation. Kaplan–Meier curves are shown in Figure 2.

### 3.4. Multivariate Cox Regression and Landmark Analyses

Results of the multivariable Cox proportional hazards regression and sensitivity analyses are presented in Table 4. After adjustment for recipient age, acute rejection within 1 year, HLA mismatching, donor sex, donor type, donor BMI, and recipient BMI, Q4 serum creatinine at one month remained an independent predictor of graft failure (HR, 1.590; 95% CI, 1.049–2.412; *p* = 0.029). Other independent risk factors included acute rejection within 1 year (HR, 1.904; 95% CI, 1.357–2.671; *p* < 0.001), deceased donor type (HR, 1.812; 95% CI, 1.309–2.509; *p* < 0.001), 4–6 HLA mismatches (HR, 1.544; 95% CI, 1.154–2.066; *p* = 0.003), female donor sex (HR, 1.391; 95% CI, 1.074–1.802; *p* = 0.013), and increasing recipient age (HR, 1.143 per 10 years; 95% CI, 1.015–1.288; *p* = 0.028). Recipient BMI and donor BMI were not independently associated with graft failure.

Additional analyses were performed in response to reviewer comments. When one-month serum creatinine was analyzed as a continuous variable, it remained significantly associated with graft failure (HR, 1.409 per 1.0 mg/dL increase; 95% CI, 1.279–1.551; *p* < 0.001). ROC analysis identified an optimal one-month serum creatinine cutoff of 1.39 mg/dL for predicting graft failure (AUC, 0.622; sensitivity, 52.6%; specificity, 65.0%). In the Cox model using this ROC-derived cutoff, creatinine above the cutoff was associated with increased risk of graft failure (HR, 1.440; 95% CI, 1.109–1.871; *p* = 0.006). Landmark analyses yielded HRs of 1.250 at 3 years and 1.161 at 5 years for Q4 versus Q1-Q3 combined; these estimates were directionally consistent but not statistically significant after restriction to patients surviving event-free to each landmark.

## 4. Discussion

The principal finding of this extended follow-up study is that serum creatinine at one month post-transplant independently stratified graft survival and renal function. Building on our previous report [18], the present analysis extends these findings by focusing on clinically relevant 3-year and 5-year endpoints and by incorporating additional reviewer-requested analyses. Recipients in the highest creatinine quartile (Q4, ≥1.52 mg/dL) had 3-year and 5-year graft survival rates of 90.3% and 86.9%, respectively, compared with 97.2% and 94.5% in Q1. Importantly, Q4 creatinine remained independently associated with graft failure even after adjustment for donor and recipient BMI.

Prior studies established the predictive value of early serum creatinine in predominantly historical cohorts, often before the adoption of modern high-risk transplantation protocols. The present cohort is distinctive in that 39.7% of recipients underwent high-risk transplantation, including ABO-incompatible transplantation, retransplantation, and highly sensitized cases requiring plasmapheresis and desensitization. Although ABO-incompatible transplantation can achieve long-term outcomes comparable to those of compatible cases with modern desensitization protocols [19,20,21], these recipients were disproportionately concentrated in higher creatinine quartiles in our cohort, reflecting the immunologic burden during the peri-transplant period. Despite this complexity, prognostic stratification by one-month creatinine remained strong and statistically significant, supporting its utility in the contemporary era.

The mechanistic basis for persistent eGFR stratification during follow-up likely reflects both nephron mass, as influenced by ischemia–reperfusion injury, donor quality [22], and early immunologic events, and cumulative injury from CNI nephrotoxicity, subclinical rejection, and progressive fibrosis. The greater eGFR decline in Q4 (−3.1%/year) than in Q1 (−1.8%/year) supports the concept that early renal reserve establishes a trajectory that is difficult to reverse with current immunosuppressive strategies [14,23]. Law et al. demonstrated that rapid eGFR deterioration (>5 mL/min/1.73 m^2^/year) during the early post-transplant period independently predicted long-term graft loss (HR, 2.17; 95% CI, 1.04–4.55) [14]. Batko et al. recently reported that baseline eGFR alone explained 87% of the variance in future 2-year eGFR, underscoring the dominant role of early renal reserve in determining the functional trajectory of the allograft [23].

Among the other independent predictors identified, acute rejection within 1 year conferred the highest risk (HR, 1.904), consistent with previous reports. Deceased-donor transplantation was also associated with significantly worse outcomes (HR, 1.812), likely reflecting the effects of cold ischemia, DGF, and marginal organ quality. Recent evidence indicates that the adverse impact of DGF on subsequent acute rejection and graft failure is broadly comparable between donation after brain death and donation after circulatory death [24,25], supporting the use of early serum creatinine as a unified prognostic tool across donor type. Importantly, despite efforts to develop novel biomarkers for long-term graft prognostication, serum creatinine-based eGFR retains superior discriminative performance (area under the curve [AUC] 0.866) compared with emerging biomarkers such as plasma neutrophil gelatinase-associated lipocalin (NGAL) (AUC 0.795) [16]. Even urinary epidermal growth factor (EGF), a promising early predictor of allograft loss, provides only modest incremental value when added to eGFR-based models [26]. The finding that Q4 creatinine remained significant after adjustment for donor type and acute rejection suggests that early creatinine captures prognostic information beyond these variables, potentially reflecting subclinical allograft injury not captured by conventional clinical endpoints.

Our findings have several clinical implications. First, serum creatinine at one month provides a simple, inexpensive, and widely available tool for risk stratification that can guide post-transplant surveillance intensity. Recipients in Q4 may warrant more frequent clinic visits, earlier biopsy-based assessment, and more aggressive management of modifiable risk factors, including blood pressure, CNI exposure, and lipid levels. Preserving graft function is important beyond transplant-specific endpoints: impaired post-transplant eGFR is independently associated with reduced physical health-related quality of life and significantly higher risks of depression (odds ratio [OR], 1.50) and anxiety (OR, 1.48) in kidney transplant recipients, as shown in a recent 2116-patient, 19-year longitudinal cohort [27]. Second, the wider prognostic gap at 5 years than at 3 years supports sustained rather than time-limited intensification of care in this high-risk group. Third, our findings suggest that future trials of novel immunosuppressive de-escalation strategies should stratify participants by early creatinine quartile to avoid baseline imbalance in graft function. From a methodological perspective, the quartile-based grouping was selected to avoid arbitrary cutoffs and evaluate a graded risk relationship; however, continuous-variable and ROC-derived cutoff analyses confirmed that the prognostic association was not solely dependent on quartile categorization. The ROC-derived cutoff of 1.39 mg/dL was close to the threshold separating the upper creatinine strata and may be clinically useful for simplified risk communication.

Several limitations should be acknowledged. First, this was a retrospective single-center study. Although the cohort was large and follow-up was extensive, selection bias and unmeasured confounding cannot be excluded. Second, immunosuppressive protocols evolved during the 13-year study period, potentially introducing era effects. Third, one-month creatinine was analyzed as a single time-point measure and may have been influenced by transient factors, such as subclinical infections or volume status. A trajectory-based approach incorporating multiple early time points, such as Bayesian eGFR curve modeling [14] or machine learning-assisted multimodal biomarker panels [15], may improve precision and enable earlier risk stratification. Fourth, data on donor-specific antibody (DSA) status at transplantation were unavailable for some recipients, precluding its inclusion as a covariate. Fifth, this cohort was from a single Korean tertiary referral center and may not be fully generalizable to populations with different genetic backgrounds or organ procurement practices. In addition, observation periods differed significantly among quartile groups; therefore, the results should be interpreted in the context of censored time-to-event analysis, and the landmark analyses should be regarded as sensitivity analyses with reduced event numbers after landmark restriction.

## 5. Conclusions

Serum creatinine level at one month post-transplant was a robust and independent predictor of graft survival and eGFR in a large contemporary KT cohort that included high-risk recipients. The association remained significant after additional adjustment for donor and recipient BMI and was confirmed when creatinine was modeled as a continuous variable and using an ROC-derived cutoff. These findings support the use of one-month creatinine as a practical risk-stratification tool to guide post-transplant surveillance intensity and identify candidates for targeted therapeutic intervention.

## Figures and Tables

**Table 1:** Baseline clinical and demographic characteristics by serum creatinine quartiles at one-month post-transplant.

Variable	Q1 (Scr < 1.0) *n* = 398	Q2 (1.0–1.23) *n* = 480	Q3 (1.23–1.52) *n* = 487	Q4 (≥1.52) *n* = 530	*p*-Value
Recipient age (years)	47.1 ± 11.4	45.7 ± 12.2	45.4 ± 11.7	44.3 ± 11.9	0.006
Donor age (years)	38.7 ± 11.4	41.2 ± 11.9	43.0 ± 11.6	46.6 ± 11.5	<0.001
Recipient BMI (kg/m^2^)	21.3 ± 3.2	22.2 ± 3.4	22.9 ± 3.0	23.3 ± 3.3	<0.001
Donor BMI (kg/m^2^)	23.6 ± 2.9	23.1 ± 3.1	23.2 ± 2.9	23.2 ± 3.0	0.057
1-month serum creatinine (mg/dL)	0.8 ± 0.1	1.1 ± 0.1	1.3 ± 0.1	2.2 ± 1.2	<0.001
1-month eGFR (mL/min/1.73 m^2^)	89.4 ± 19.6	68.7 ± 10.2	58.0 ± 8.6	39.5 ± 13.2	<0.001
3-year eGFR (mL/min/1.73 m^2^)	80.4 ± 23.8	70.7 ± 19.1	66.4 ± 16.2	57.1 ± 17.3	<0.001
5-year eGFR (mL/min/1.73 m^2^)	79.0 ± 25.3	69.4 ± 20.9	66.0 ± 18.5	58.2 ± 19.1	<0.001
Recipient sex, female	324 (81.4)	244 (50.8)	123 (25.3)	101 (19.1)	<0.001
Donor sex, female	148 (37.2)	238 (49.6)	271 (55.6)	289 (54.5)	<0.001
Donor type: living related	221 (55.5)	248 (51.7)	223 (45.8)	197 (37.2)	<0.001
Donor type: living unrelated	92 (23.1)	129 (26.9)	152 (31.2)	139 (26.2)	
Donor type: deceased donor	85 (21.4)	103 (21.5)	112 (23.0)	194 (36.6)	
ABO-incompatible	49 (12.3)	59 (12.3)	52 (10.7)	39 (7.4)	0.035
Re-transplantation	52 (13.1)	45 (9.4)	40 (8.2)	33 (6.2)	0.004
Pre-transplant plasmapheresis/desensitization	84 (21.1)	82 (17.1)	65 (13.3)	55 (10.4)	<0.001
HLA mismatch: zero	43 (10.8)	53 (11.0)	53 (10.9)	52 (9.8)	0.032
HLA mismatch: 1–3	249 (62.6)	287 (59.8)	259 (53.2)	291 (54.9)	
HLA mismatch: 4–6	106 (26.6)	140 (29.2)	175 (35.9)	187 (35.3)	
Acute rejection within 1 year	3 (0.8)	21 (4.4)	36 (7.4)	75 (14.2)	<0.001
Delayed graft function/ATN	7 (1.8)	24 (5.0)	34 (7.0)	101 (19.1)	<0.001

Abbreviations: Scr, serum creatinine; eGFR, estimated glomerular filtration rate; HLA, human leukocyte antigen. Observation periods differed among groups (*p* < 0.001): the median follow-up durations were 77.5 months in Q1, 96.0 months in Q2, 95.0 months in Q3, and 102.0 months in Q4. Therefore, time-to-event analyses and landmark analyses were used to account for censored observations and unequal follow-up durations.

**Table 2:** Serial eGFR by serum creatinine quartiles at 1, 3, and 5 years post-transplant.

Time Point	Q1 (Scr < 1.0)	Q2 (1.0–1.23)	Q3 (1.23–1.52)	Q4 (≥1.52)	*p*-Value
1-month post-Tx (Scr, mg/dL)	0.80 ± 0.11	1.08 ± 0.07	1.34 ± 0.08	2.21 ± 1.18	<0.001
1-month eGFR (mL/min/1.73 m^2^)	89.4 ± 19.6	68.7 ± 10.2	58.0 ± 8.6	39.5 ± 13.2	<0.001
1-year eGFR (mL/min/1.73 m^2^)	80.3 ± 19.3	67.7 ± 17.2	61.3 ± 15.2	52.0 ± 15.0	<0.001
3-year eGFR (mL/min/1.73 m^2^)	80.4 ± 23.8	70.7 ± 19.1	66.4 ± 16.2	57.1 ± 17.3	<0.001
5-year eGFR (mL/min/1.73 m^2^)	79.0 ± 25.3	69.4 ± 20.9	66.0 ± 18.5	58.2 ± 19.1	<0.001

Abbreviations: Tx, transplantation; Scr, serum creatinine; eGFR, estimated glomerular filtration rate.

**Table 3:** Three-year and 5-year graft survival outcomes by serum creatinine quartile.

	Q1 (Scr < 1.0) *n* = 398	Q2 (1.0–1.23) *n* = 480	Q3 (1.23–1.52) *n* = 487	Q4 (≥1.52) *n* = 530	*p*-Value
Graft failure events, n (%)	33 (8.3)	52 (10.8)	59 (12.1)	107 (20.2)	<0.001
3-year graft survival rate (%)	97.2	96.2	95.3	90.3	<0.001
5-year graft survival rate (%)	94.5	94.6	92.5	86.9	<0.001
Patients at risk at 3 years, n	362	421	422	438	
Patients at risk at 5 years, n	318	374	371	356	
Median follow-up (months), median (IQR)	77.5 (48.2–110.8)	96.0 (56.0–139.0)	95.0 (56.0–130.0)	102.0 (56.0–139.0)	<0.001

Abbreviations: Scr, serum creatinine; IQR, interquartile range.

**Table 4:** Multivariable Cox proportional hazards regression for graft failure and 3-year and 5-year landmark analyses.

Variable	B	*p*-Value	HR	95% CI (Lower)	95% CI (Upper)
Serum creatinine at one-month post-Tx					
Q1 (<1.0 mg/dL; reference)	0	-	1.000	-	-
Q2 (1.0–1.23 mg/dL)	0.029	0.898	1.029	0.661	1.603
Q3 (1.23–1.52 mg/dL)	0.078	0.729	1.081	0.697	1.676
Q4 (≥1.52 mg/dL)	0.464	0.029	1.590	1.049	2.412
Recipient age (per 10 years)	0.134	0.028	1.143	1.015	1.288
Acute rejection within 1 year	0.644	<0.001	1.904	1.357	2.671
HLA mismatching (4–6 Ag)	0.435	0.003	1.544	1.154	2.066
Donor sex, female	0.330	0.013	1.391	1.074	1.802
Donor type, living unrelated (ref: living related)	−0.027	0.887	0.974	0.675	1.404
Donor type, deceased (ref: living related)	0.595	<0.001	1.812	1.309	2.509
Recipient BMI	0.007	0.742	1.007	0.967	1.048
Donor BMI	0.011	0.612	1.011	0.970	1.053
Continuous creatinine (per 1.0 mg/dL increase)	0.343	<0.001	1.409	1.279	1.551
ROC-derived cutoff ≥ 1.39 mg/dL	0.365	0.006	1.440	1.109	1.871
3-year landmark: Q4 vs. Q1–Q3	0.223	0.212	1.250	0.881	1.774
5-year landmark: Q4 vs. Q1–Q3	0.149	0.482	1.161	0.766	1.759

Abbreviations: B, regression coefficient; HR, hazard ratio; CI, confidence interval; Tx, transplantation; HLA, human leukocyte antigen; ref, reference. The upper panel (Q1–Q4 through Donor BMI) represents the primary multivariable Cox proportional hazards model. Continuous creatinine and ROC-derived cutoff rows are from independent sensitivity analyses using separate Cox models with the same covariates. Landmark analyses (3-year and 5-year) were conducted as separate Cox models restricted to recipients who had not experienced graft failure or death by the respective landmark time point.

## Data Availability

Data available on request due to restrictions (e.g., privacy, legal or ethical reasons).

## Abbreviations

The following abbreviations are used in this manuscript:

KT	kidney transplantation
HLA	human leukocyte antigen
DGF	delayed graft function
CNI	calcineurin inhibitor
eGFR	estimated glomerular filtration rate
MDRD	Modification of Diet in Renal Disease
SD	standard deviation
ANOVA	analysis of variance
HR	hazard ratio
CI	confidence interval
NGAL	neutrophil gelatinase-associated lipocalin
AUC	area under the curve
EGF	epidermal growth factor
OR	odds ratio
DSA	donor-specific antibody
BMI	body mass index
